# Noradrenergic stimulation of α_1_ adrenoceptors in the medial prefrontal cortex mediates acute stress-induced facilitation of seizures in mice

**DOI:** 10.1038/s41598-023-35242-0

**Published:** 2023-05-19

**Authors:** Kazuhei Niitani, Shiho Ito, Shintaro Wada, Shoma Izumi, Naoya Nishitani, Satoshi Deyama, Katsuyuki Kaneda

**Affiliations:** Laboratory of Molecular Pharmacology, Institute of Medical, Pharmaceutical and Health Sciences, Kanazawa University, Kanazawa, 920-1192 Japan

**Keywords:** Neuroscience, Neurology

## Abstract

Stress is one of the critical facilitators for seizure induction in patients with epilepsy. However, the neural mechanisms underlying this facilitation remain poorly understood. Here, we investigated whether noradrenaline (NA) transmission enhanced by stress exposure facilitates the induction of medial prefrontal cortex (mPFC)-originated seizures. In mPFC slices, whole-cell current-clamp recordings revealed that bath application of picrotoxin induced sporadic epileptiform activities (EAs), which consisted of depolarization with bursts of action potentials in layer 5 pyramidal cells. Addition of NA dramatically shortened the latency and increased the number of EAs. Simultaneous whole-cell and field potential recordings revealed that the EAs are synchronous in the mPFC local circuit. Terazosin, but not atipamezole or timolol, inhibited EA facilitation, indicating the involvement of α_1_ adrenoceptors. Intra-mPFC picrotoxin infusion induced seizures in mice in vivo. Addition of NA substantially shortened the seizure latency, while co-infusion of terazosin into the mPFC inhibited the effect of NA. Finally, acute restraint stress shortened the latency of intra-mPFC picrotoxin infusion-induced seizures, whereas prior infusion of terazosin reversed this stress-induced shortening of seizure latency. Our findings suggest that stress facilitates the induction of mPFC-originated seizures via NA stimulation of α_1_ adrenoceptors.

## Introduction

Epilepsy is a chronic neurological disorder and affects approximately 1% of the world’s population. Excessive synchronous neuronal activities induce characteristic repetitive seizures. The loss of γ-amino butyric acid (GABA)-ergic neurons and abnormal GABA receptors are observed in animal models and patients with epilepsy^1–3^. Additionally, blockade of GABA_A_ receptors induces epileptiform activity (EA) in in vivo and in vitro^4,5^. Therefore, GABAergic dysfunction plays a critical role in inducing the imbalance between excitation and inhibition, leading to epileptic seizures.

Stress is one of the frequently self-reported precipitants for seizure induction in patients with epilepsy^6–9^. Stress hormones, such as cortisol and corticosterone, have been reported to be involved in stress-induced epileptic seizures^10–12^. However, it remains unclear how stress triggers seizures. The locus coeruleus (LC) is the source of noradrenaline (NA) in the brain and sends efferent projections to numerous areas of the brain, including the neocortex, hippocampus, and amygdala^13^. Stress activates LC neurons, leading to increased NA release in these brain regions^14–19^. The effect of NA on epileptic seizures has been mainly studied in the limbic system, such as the hippocampus and amygdala that are known for frequent seizure origin. NA is generally considered to have an anticonvulsive effect in those regions^20^. In contrast, the effect of NA on frontal lobe epilepsy (FLE) has not been well studied, even though FLE is the second most common type of refractory focal seizure after temporal lobe epilepsy (TLE)^21^. The ventromedial prefrontal cortex (vmPFC) is also a known focus of pharmacoresistant epilepsy^22^. Stress enhances NA release in the medial prefrontal cortex (mPFC) in rodents^15–17^, which corresponds to the caudal region of the vmPFC in humans^23^. In addition, we and several other researchers have shown that NA excites mPFC layer 5 (L5) pyramidal cells via α_1_ and β adrenoceptors in rodents^24–26^. Thus, it is hypothesized that the excitatory effect of NA may contribute to the induction of abnormal, excessive synchronous neuronal activities in epileptic seizures. Therefore, in this study, we investigated whether increased NA levels in the mPFC are involved in the induction of epileptic seizures caused by stress exposure using behavioral and electrophysiological approaches.

## Methods

### Animals

Male and female C57BL/6J mice (4–8 weeks of age, *n* = 55) were used for electrophysiological experiments, and male C57BL/6J mice (6–8 weeks of age, *n* = 71) were used for behavioral experiments. Mice were maintained at a constant ambient temperature (22 ± 1 °C) and under a 12-h light/dark cycle, with food and water available ad libitum as described previously^26–28^. All experiments were conducted in accordance with the National Institutes of Health guidelines and the ARRIVE guidelines and performed with the approval of the Institutional Animal Care and Use Committee at Kanazawa University. All efforts were made to minimize the suffering and number of animals used in this study.

### Drugs

In electrophysiological experiments, l-noradrenaline bitartrate monohydrate (NA; Tokyo Chemical Industry, Tokyo, Japan), CNQX (Alomone Labs, Jerusalem, Israel), dl-AP-5 (AP-5; Tocris Bioscience, Bristol, UK), terazosin hydrochloride (Sigma, St. Louis, MO, USA), atipamezole hydrochloride (Wako, Osaka, Japan), timolol maleate (Sigma), and phenylephrine hydrochloride (Wako) were dissolved in distilled water. Picrotoxin (Sigma) was dissolved in dimethyl sulfoxide. These drug solutions were diluted with standard Ringer’s solution before use. Kynurenic acid hydrate (KYNA; Tokyo Chemical Industry) was dissolved in standard Ringer’s solution. In behavioral experiments, picrotoxin, NA, and terazosin (Tocris) were dissolved in phosphate-buffered saline (PBS). Picrotoxin injection into the cortex has been reported to elicit seizures^29^. The doses of drugs were chosen based on the previous studies^26,30–32^.

### Slice preparation and electrophysiology

Procedures for preparing mPFC slices and electrophysiological recordings were similar to our previous studies^26,33,34^. Mice were anesthetized with isoflurane and decapitated. The brains were then dissected and submerged in ice-cold modified Ringer’s solution (containing the following [in mM]: choline chloride, 125; KCl, 4.0; NaH_2_PO_4_, 1.25; MgCl_2_, 7.0; CaCl_2_, 0.5; NaHCO_3_, 26; glucose, 20; ascorbate, 1.0; and pyruvate, 3.0) and bubbled with 95% O_2_/5% CO_2_ (pH 7.4). Coronal slices (250 μm thick) of the mPFC were cut using a microslicer (VT1200S; Leica, Wetzlar, Germany) and incubated at 32–34 °C for 15–30 min in standard Ringer’s solution (containing the following [in mM]: NaCl, 125; KCl, 2.5; NaH_2_PO_4_, 1.25; MgCl_2_, 0.9; CaCl_2_, 2.0; NaHCO_3_, 26; and glucose, 25) and bubbled with 95% O_2_/5% CO_2_ (pH 7.4). Next, the slices were transferred to standard Ringer’s solution at room temperature and then mounted in a recording chamber on an upright microscope (BX-51WI; Olympus, Tokyo, Japan). The chamber was continuously superfused with standard Ringer’s solution at a flow rate of 2–2.5 mL/min. In some experiments, mPFC-isolated slices were prepared by trimming the coronal slices.

Whole-cell patch-clamp recordings were obtained from mPFC L5 pyramidal cells by visually controlling patch pipettes, which were prepared from borosilicate glass capillaries and filled with an internal solution. For whole-cell voltage- and current-clamp recordings, we used internal solutions containing (in mM) CsOH, 150; CsCl, 5.0; MgCl_2_, 2.0; Na_2_ATP, 4.0; Na_3_GTP, 0.3; EGTA, 10; HEPES, 10; QX-314, 3.0 (pH 7.3 with gluconic acid) and KOH, 150; KCl, 10; MgCl_2_, 2.0; Na_2_ATP, 2.0; Na_3_GTP, 0.3; EGTA, 0.2; HEPES, 10; spermine, 0.1 (pH 7.3 with gluconic acid), respectively. In order to identify and stain the recorded neurons, Alexa Fluor 594 (0.02 mM; Thermo Fisher Scientific, Waltham, MO, USA) and biocytin (1–3 mg/mL; Sigma) were dissolved in the corresponding internal solution. The resistance of the electrodes was 3–9 MΩ in Ringer’s solution. All recordings were performed at 32–34 °C.

For recording evoked inhibitory postsynaptic currents (eIPSCs) using the whole-cell voltage-clamp technique, the membrane potentials were held at + 10 mV in the presence of a glutamate receptor antagonist KYNA (2 mM), and electrical stimulations were applied as cathodal square-wave pulses of 200-μs duration with an intensity of up to 30 μA using a glass electrode that was located in layer 2/3 of the mPFC and filled with normal Ringer’s solution. The eIPSC amplitudes were measured in periods of 0–30 s before the end of the drug applications. The picrotoxin effects were evaluated by comparing the average values of these periods.

For whole-cell current-clamp recordings, membrane potentials were recorded without holding current injection. The generation of EAs, defined as prolonged depolarization accompanied with three or more burst firings, was recorded for 15 min (time 0 = start of drug application). EA latency was measured as the time of the induction of the first EA after drug application. If EA was not induced within 15 min, the latency was calculated as 15 min. The duration and number of burst firings of an EA were measured on the first EA in periods of 0–5 min and 10–15 min after drug application (picrotoxin, picrotoxin + NA, or picrotoxin + phenylephrine). In pharmacological experiments, EA frequency was measured in periods of 0–3 min before and 2–5 min after the application of antagonists. In some experiments, whole-cell current-clamp and field potential recordings were obtained simultaneously. The field potential recordings were obtained using a glass electrode placed in the mPFC L5 and containing 2.5 M NaCl with a resistance of 1–3 MΩ in Ringer’s solution.

Data were amplified with a Multiclamp 700B (Molecular Devices, Foster City, CA, USA) and low-pass-filtered at 3 kHz. Additionally, field potential data were high-pass-filtered at 0.1 Hz. Voltage-clamp, current-clamp, and simultaneous recording data were then digitized at 20, 6, and 10 kHz with an A/D interface (Digidata 1440A; Molecular Devices), respectively, and stored on a computer using a Clampfit 10.5 (Molecular Devices). Field potential data were further low-pass-filtered at 30 Hz with the Clampfit 10.5. The correlation between EAs and field potentials was evaluated by cross-correlation analysis. The cross-correlogram was constructed using onset time points of the first peaks of EAs and the peaks of negative shifts of field potentials. The calculation was based on the previously described methods^35^ and performed using the ‘scipy.signal.correlate’ function in the SciPy library in Python. A bin width of 300 ms was used.

### Histochemistry

Histochemical examinations of recorded neurons were implemented as described previously^26,33,34^. After the recordings, the slices were fixed overnight in 4% paraformaldehyde in 0.1 M PBS (pH 7.4) at 4 °C. After three rinses with 0.05 M PBS, the slices were then incubated in 0.6% H_2_O_2_ in methanol for 30 min at room temperature to eliminate endogenous peroxidase activity, followed by 3 h of incubation in an avidin–biotin-peroxidase complex using a R.T.U. ABC Reagent (Vector laboratories, Burlingame, CA, USA) diluted fourfold in PBS containing 0.3% Triton X-100. The slices were then rinsed in 0.05 M Tris–HCl (pH 7.5) and incubated in 0.05% 3,3′-diaminobenzidine (DAB; Nacalai Tesque, Kyoto, Japan) solution. Next, the biocytin-filled cells were stained brown, and the reaction was stopped by rinsing with 0.05 M PBS. The biocytin-labeled cells were identified under a microscope (BZ-9000; Keyence, Osaka, Japan). L5 pyramidal cells were identified according to the following characteristics: the depth of the cells from the midline (350–550 μm) and shapes of the soma and apical dendrites.

### Surgery and intra-mPFC infusion

Intra-mPFC infusions were performed as previously described with slight modification^36–38^. Mice were anesthetized with chloral hydrate (400 mg/kg, i.p.) and implanted with 25-gauge stainless-steel guide cannulae (o.d., 0.51 mm; i.d., 0.26 mm) above the mPFC (1.8 mm rostral, ± 1.3 mm lateral, − 1.5 mm ventral to bregma, at a 20° angle from the vertical axis in the mediolateral plane; bilateral)^39^. After surgery, the mice were housed individually and allowed to recover for at least 5 days. For intra-mPFC infusions, 33-gauge stainless-steel infusion cannulae (o.d., 0.2 mm; i.d., 0.08 mm) were inserted bilaterally into the guide cannula, with the infusion cannulae protruded 1.1 mm from the tip of the guide cannula to reach the mPFC. The drugs (or vehicle) were administered bilaterally in a volume of 0.2 μL/side and at a rate of 0.2 μL/min. After the infusion, the infusion cannulae were kept in place for an additional 1 min after intra-mPFC infusion to prevent backflow.

### Behavioral seizure observation

All mice were handled for 2 consecutive days, followed by habituation to a transparent testing chamber (32 cm × 22 cm × 13.5 cm; Natsume Seisakusho, Tokyo, Japan) for 30 min. On day 1, each mouse was administered a bilateral injection of picrotoxin (0.1 nmol/side) into the mPFC, placed in the testing chamber for 1800s (30 min), and videotaped. On day 2, the effects of intra-mPFC infusion of NA were tested in each mouse by administering a bilateral injection of either picrotoxin (0.1 nmol/side) only, picrotoxin + terazosin (picrotoxin: 0.1 nmol/side, terazosin: 7.5 nmol/side), picrotoxin + NA (picrotoxin: 0.1 nmol/side, NA: 10 nmol/side), or picrotoxin + NA + terazosin (picrotoxin: 0.1 nmol/side, NA: 10 nmol/side, terazosin: 7.5 nmol/side) into the mPFC. The mice were then immediately placed in the testing chamber for 1800s and videotaped. On day 2, the effects of acute restraint stress were tested in each mouse by administering a bilateral injection of either vehicle (PBS) or terazosin (7.5 nmol/side) into the mPFC, followed by 20 min immobilization using a plastic bag (DecapiCones; Braintree Scientific, Braintree, MA, USA). Immediately after the restraint stress, each mouse was administered a bilateral injection of picrotoxin (0.1 nmol/side) into the mPFC, placed in the testing chamber for 1800s, and videotaped.

Behavioral seizures were classified on the basis of the modified Racine scale^40,41^: stage 1, absence‐like immobility; stage 2, hunching with facial or manual automatisms; stage 3, rearing with facial or manual automatisms and forelimb clonus; stage 4, repeated rearing with continuous forelimb clonus and falling; and stage 5, generalized tonic–clonic convulsions with lateral recumbence or jumping and wild running followed by generalized convulsions. Stage 1 and 2 were reported as non‐convulsive seizures with no clear motor component, whereas stage 3 and above were convulsive motor seizures^41^. Thus, we measured the latency of stage 3 seizures.

### Histology

After the behavioral tests, histological analyses were performed to confirm the infusion sites in the mPFC using the methods reported in our previous studies^26,27,36–38^. Briefly, the mice were decapitated, and the brains were rapidly dissected and frozen in powdered dry ice. Coronal Sects. (50 μm thick) were prepared on a cryostat, thaw-mounted onto slides, stained with thionin, and examined under a microscope (BZ-9000). Data from mice with incorrect infusion placements (n = 22) were excluded from statistical analyses.

### Statistical analyses

Data are expressed as mean ± standard error of the mean. The data were compared using Student’s *t*-test or paired *t*-test when comparing two groups, one-way repeated measures ANOVA with post hoc Holm–Sidak’s multiple comparison test or two-way repeated measures ANOVA with post hoc Bonferroni’s multiple comparison test when comparing more than two groups. All analyses were performed using Prism 6 (GraphPad Software, La Jolla, CA, USA). We also confirmed that all experiments conducted in this study had adequate statistical power (greater than 0.9) using G*Power software 3.1^42^. Statistical significance was set at *P* value < 0.05.

## Results

### NA promotes induction of EAs in mPFC L5 pyramidal cells under the suppression of GABA_A_ receptor-mediated inhibition in vitro

**Figure 1:**
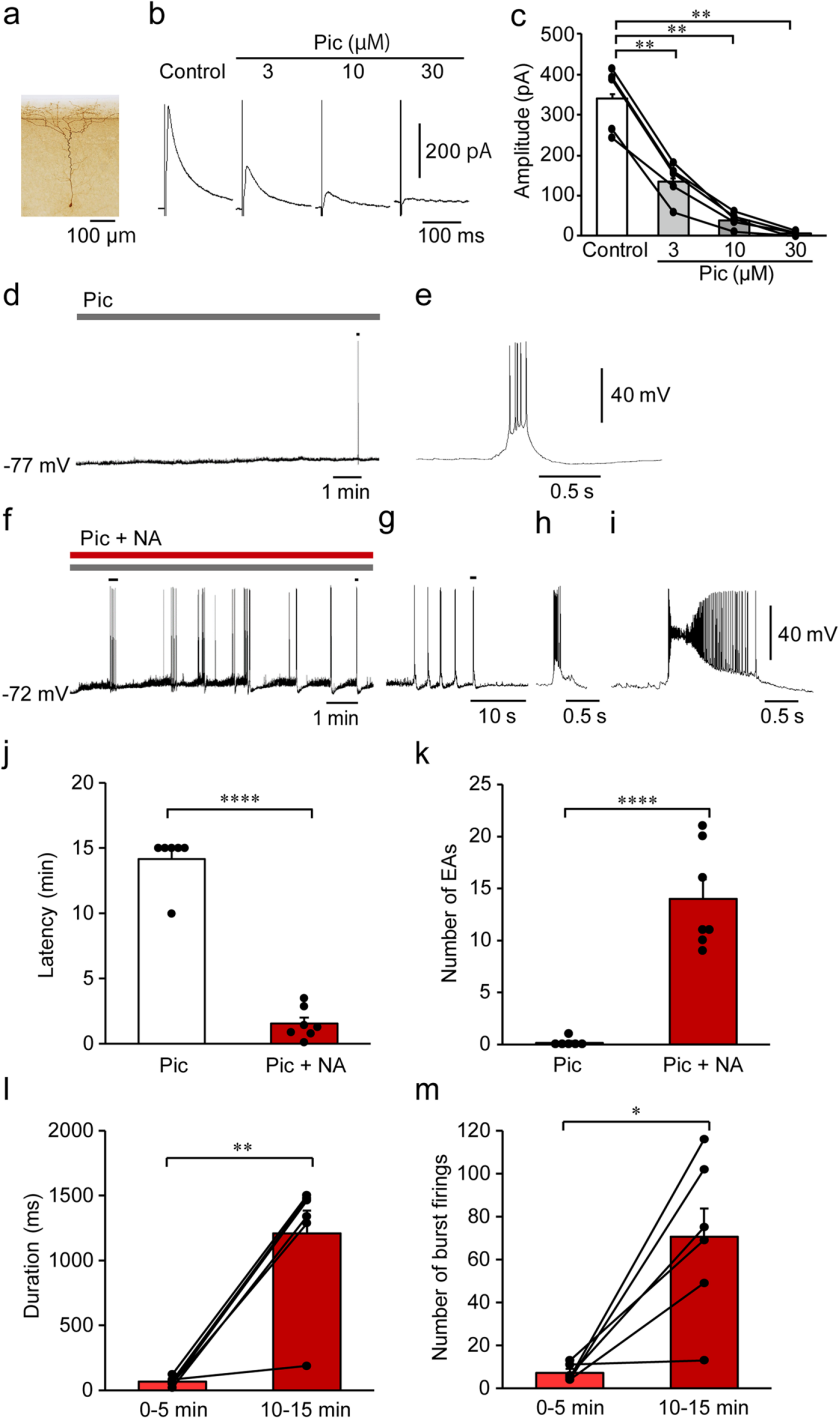
Noradrenaline (NA) promotes the induction of epileptiform activity (EA) in layer 5 pyramidal cells of the medial prefrontal cortex under the suppression of inhibitory synaptic transmission. (**a**) Representative micrograph of a recorded pyramidal cell. (**b**) Representative traces showing the effects of picrotoxin (Pic) on evoked inhibitory postsynaptic currents (eIPSCs). (**c**) Summary graph showing the effects of Pic on eIPSCs (*n* = 5 from 4 mice). **P < 0.01 (one-way ANOVA with post hoc Holm–Sidak’s test). (**d**, **f**) Representative traces of membrane potentials after the application of Pic (**d**) and Pic + NA (**f**). (**e**, **g**, **h, i**) Expanded traces of a mark in **d** (**e**), marks at short (**g**) and long (**i**) times after the application of NA in **f**, and a mark in **g** (**h**). (**j**, **k**) Summary graphs of EA latency (**j**) and number of EAs (**k**) after the application of Pic (*n* = 6 from 4 mice) or Pic + NA (*n* = 7 from 7 mice). (**l**, **m**) Summary graphs of changes in duration (**l**) and number of burst firings (**m**) in EAs after the application of NA (*n* = 6 from 6 mice). *P < 0.05, **P < 0.01, ****P < 0.0001 (Student’s *t*-test or paired *t*-test). Data are expressed as mean ± standard error of the mean.

We first examined the effects of picrotoxin, a GABA_A_ receptor antagonist, on eIPSCs in mPFC L5 pyramidal cells using in vitro whole-cell current clamp recordings (Fig. 1a). Bath application of picrotoxin (3, 10, and 30 µM) reduced eIPSC amplitude in a concentration-dependent manner, with 30 µM picrotoxin demonstrating almost complete inhibition of eIPSC (control: 340.8 ± 35.7 pA; 3 µM: 135.7 ± 21.6 pA; 10 µM: 39.1 ± 8.2 pA; and 30 µM: 5.2 ± 3.3 pA; control vs. 3 µM, *P* = 0.0035; control vs. 10 µM, *P* = 0.0034; and control vs. 30 µM, *P* = 0.0035, *n* = 5, one-way repeated measures ANOVA with post hoc Holm–Sidak’s test, Fig. 1b,c). Thus, we used 30 µM picrotoxin to reproduce an epileptiform state^5,43^ in the following experiments.

Bath application of picrotoxin induced an EA, which consisted of a prolonged depolarization, the so-called paroxysmal depolarization shift (PDS)^44^, accompanied with more than 3 burst firings in 1 of 6 cells tested (Fig. 1d,e). The first EA was observed at least 7 min after the application of picrotoxin. The number of EAs generated during the 15 min recording (time 0 = start of picrotoxin application) was 1. EA was not induced during the 15 min recording in the remaining 5 cells. Picrotoxin did not significantly affect resting membrane potentials (control, − 73.15 ± 1.06 mV vs. picrotoxin, − 73.06 ± 1.10 mV, *n* = 7, *t*_6_ = 0.3192, *P* = 0.7604, paired *t*-test). These findings indicate that picrotoxin application alone induces EA quite sporadically.

Next, we examined the effects of NA on the generation of EA. Simultaneous bath application of picrotoxin + NA (10 µM) immediately induced depolarization (control, − 73.15 ± 1.06 mV vs. picrotoxin + NA, − 71.09 ± 1.25 mV, n = 7, *t*_6_ = 3.543, *P* = 0.0122, paired *t*-test) with/without firings as previously reported^26^. Soon after the depolarization, EAs were rapidly induced with a latency of 1.6 ± 0.5 min (*n* = 7), which was significantly shorter than that of the EAs observed in the presence of picrotoxin alone (14.2 ± 0.84 min, *n* = 6; picrotoxin vs. picrotoxin + NA,* t*_11_ = 13.80, *P* < 0.0001, Student’s *t*-test, Fig. 1f,j). The number of EAs after picrotoxin + NA (14.0 ± 1.9) application was significantly larger than that after picrotoxin alone (0.167 ± 0.17; picrotoxin vs. picrotoxin + NA,* t*_11_ = 6.760, *P* < 0.0001, Student’s *t*-test, Fig. 1f,k). Additionally, the duration of PDS and number of burst firings in an EA gradually increased over time (duration: 65.65 ± 14.4 ms at 0–5 min after picrotoxin + NA application vs. 1209.80 ± 207.2 ms at 10–15 min after picrotoxin + NA application, *n* = 6,* t*_5_ = 5.469, *P* = 0.0028, Fig. 1g,h,i,l; number of burst firings: 7.2 ± 1.6 at 0–5 min after picrotoxin + NA application vs. 70.7 ± 15.1 at 10–15 min after picrotoxin + NA application, *t*_5_ = 3.975, *P* = 0.0106, Fig. 1g,h,i,m). These findings demonstrate that NA dramatically promotes the induction and enhances the strength of EAs in mPFC L5 pyramidal cells under suppressed inhibitory synaptic transmission.

### EAs are induced synchronously via glutamatergic transmission in the mPFC local circuit

**Figure 2:**
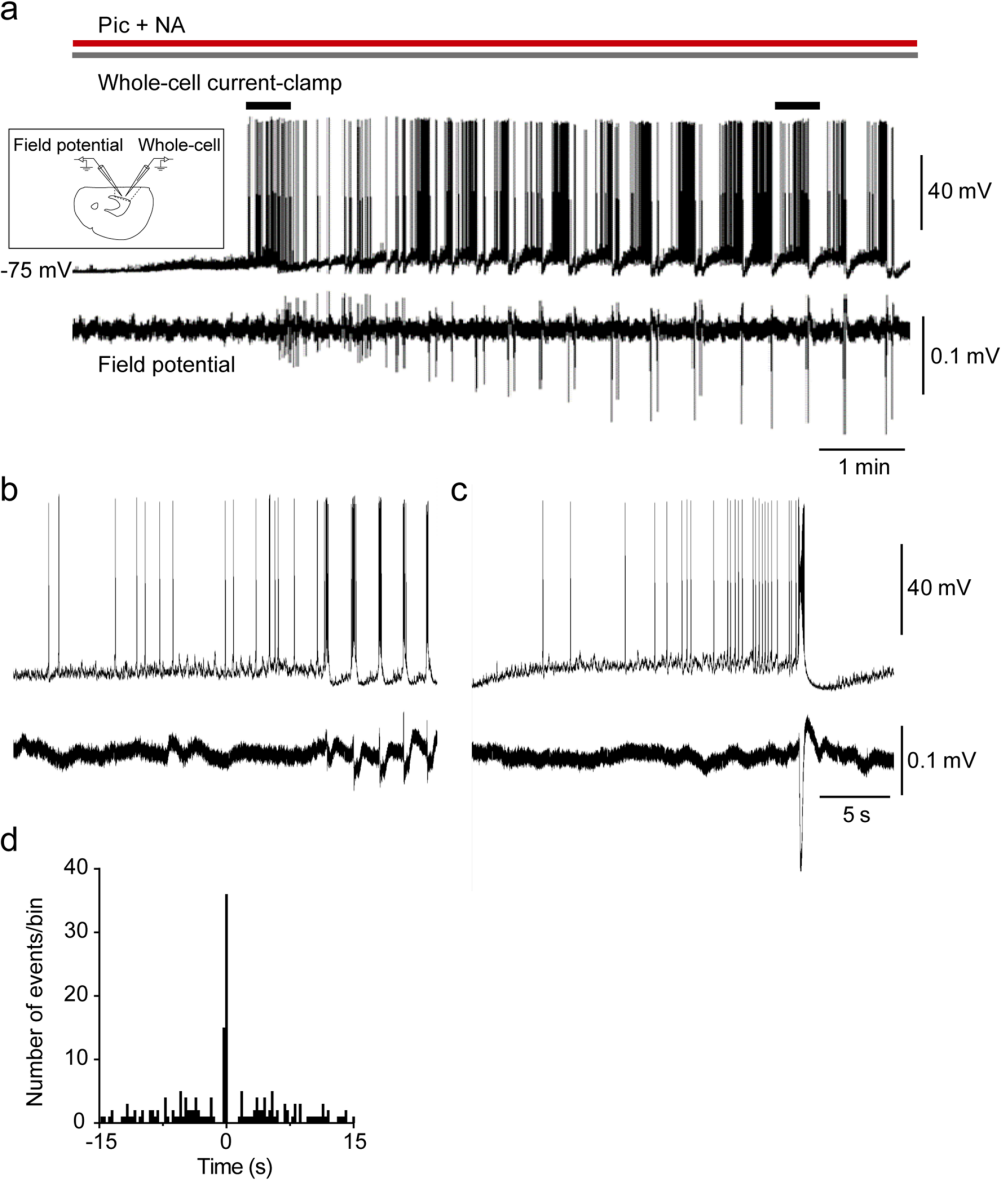
Epileptiform activities are induced synchronously in the medial prefrontal cortex. (**a**) Representative traces of membrane potentials and population spikes from simultaneous whole-cell and field potential recordings after the application of picrotoxin (Pic) + noradrenaline (NA). (inset) Schematic image of whole-cell and field potential recordings. (**b**, **c**) Expanded traces of marks at short (**b**) and long (**c**) times after the application of Pic + NA in (**a**). (**d**) Cross-correlogram from the traces in (**a**) (bin width = 300 ms).

Using simultaneous whole-cell current-clamp and field potential recordings, we next investigated whether EAs are synchronous. Clear population activities were not observed shortly after the application of picrotoxin + NA, which is the time at which small EAs were generated as described above. However, several minutes later, gradual augmentation of population activities and emergence of clear negative potentials, which were time-locked to enlarged EAs, were observed (*n* = 6, Fig. 2a,b,c). To investigate the temporal relationship between the onset times of EAs and negative shifts in the field potentials more clearly, we conducted cross-correlation analysis. The highest peak was detected at time 0 with a bin width of 300 ms (Fig. 2d), indicating that EAs and negative shifts of field potentials occurred within 300 ms. These results indicate that EAs are synchronous in the mPFC.

**Figure 3:**
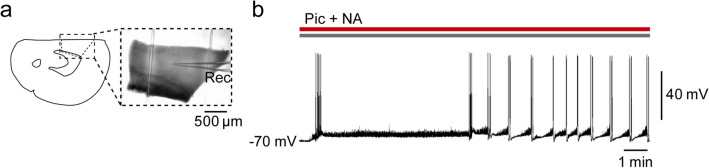
Epileptiform activities are induced in medial prefrontal cortex (mPFC)-isolated slices. (**a**) Representative image of whole-cell current-clamp recording of an mPFC-isolated slice. Rec, recording electrode. (**b**) Representative trace of membrane potentials from whole-cell current-clamp recording in an mPFC-isolated slice after the application of picrotoxin (Pic) + noradrenaline (NA) (*n* = 6 from 6 mice).

We next addressed whether EAs are induced in the mPFC local circuits using mPFC-isolated slices (Fig. 3a). Whole-cell current-clamp recordings revealed that EAs were still induced in these slices after picrotoxin + NA application (6 of 6 cells; Fig. 3b), indicating that mPFC local circuits are sufficient for inducing EAs.

Previous studies reported that NA enhances glutamatergic transmission in mPFC L5 pyramidal cells^25,26^. Because EAs were induced synchronously in the mPFC local circuit, we hypothesized that the mutual activation of mPFC pyramidal cells through glutamatergic transmission may contribute to the NA-induced facilitation of EAs. Addition of CNQX (10 µM), an AMPA receptor antagonist, to picrotoxin + NA completely suppressed the generation of EAs (picrotoxin + NA, 1.8 ± 0.3 events/min vs. picrotoxin + NA + CNQX, 0.0 ± 0.0 events/min, *n* = 6, *t*_5_ = 5.219, *P* = 0.0034, paired *t*-test, supplementary Fig. S1a,b). Similarly, the addition of AP-5 (50 µM), an NMDA receptor antagonist, also significantly reduced EA generation (picrotoxin + NA, 1.8 ± 0.4 events/min vs. picrotoxin + NA + AP-5, 0.3 ± 0.2 events/min, *n* = 8, *t*_7_ = 4.549, *P* = 0.0026, paired *t*-test, supplementary Fig. S1c,d). These results reveal that both AMPA and NMDA receptors play a critical role in NA-induced promotion of EAs.

### Alpha 1 adrenoceptors mediate NA-induced facilitation of EAs

**Figure 4:**
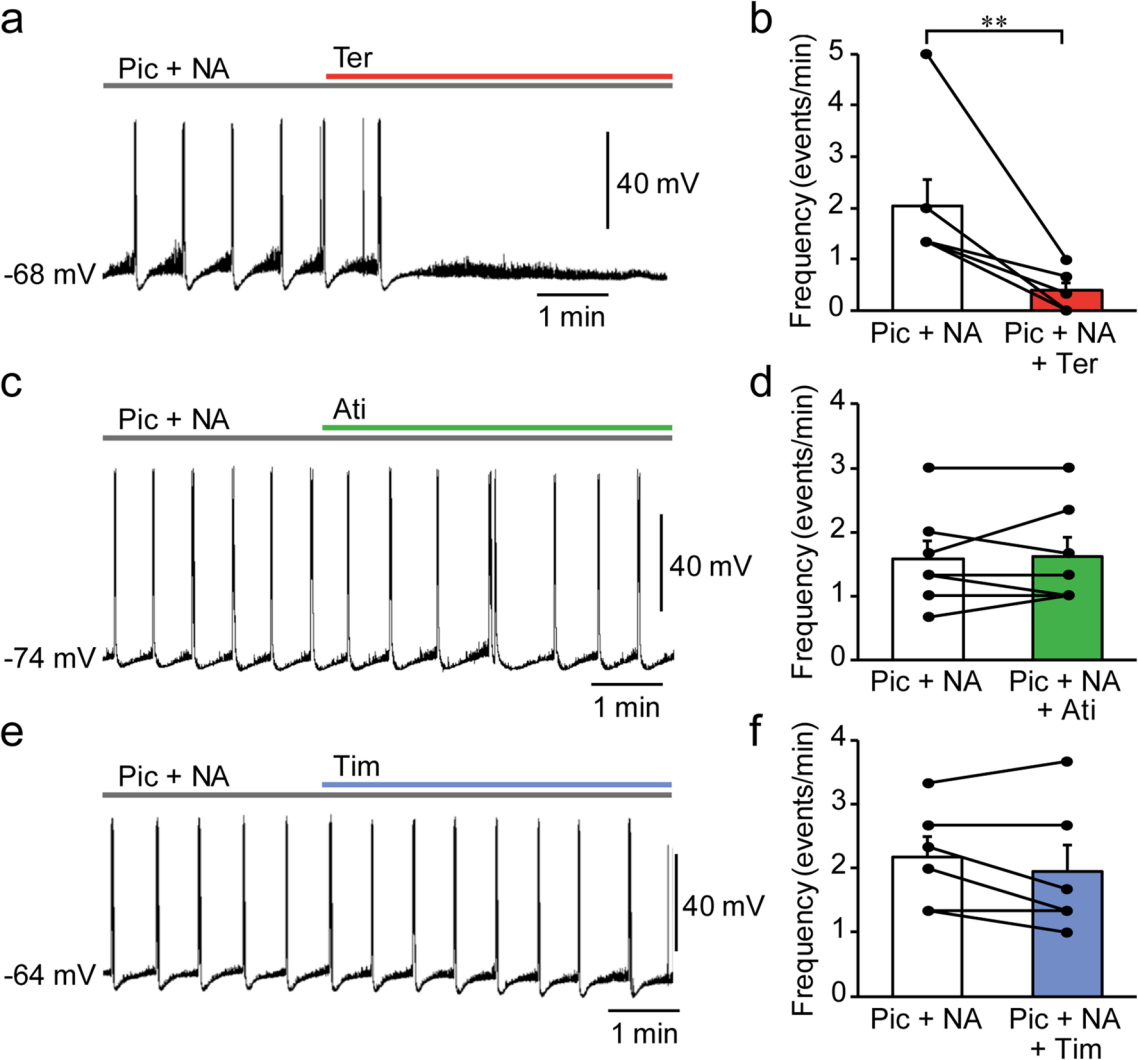
Blockade of α_1_ adrenoceptors inhibits noradrenaline (NA)-induced epileptiform activities (EAs). (**a**, **c**, **e**) Representative traces of membrane potentials before and after the addition of terazosin (Ter, **a**), atipamezole (Ati, **c**), and timolol (Tim, **e**) to Pic + NA. (**b**, **d**, **f**) Summary graphs showing the effects of Ter (**b**, *n* = 7 from 7 mice), Ati (**d**, *n* = 7 from 6 mice), and Tim (**f**, *n* = 6 from 5 mice) on EA frequency. **P < 0.01 (paired *t*-test). Data are expressed as mean ± standard error of the mean.

**Figure 5:**
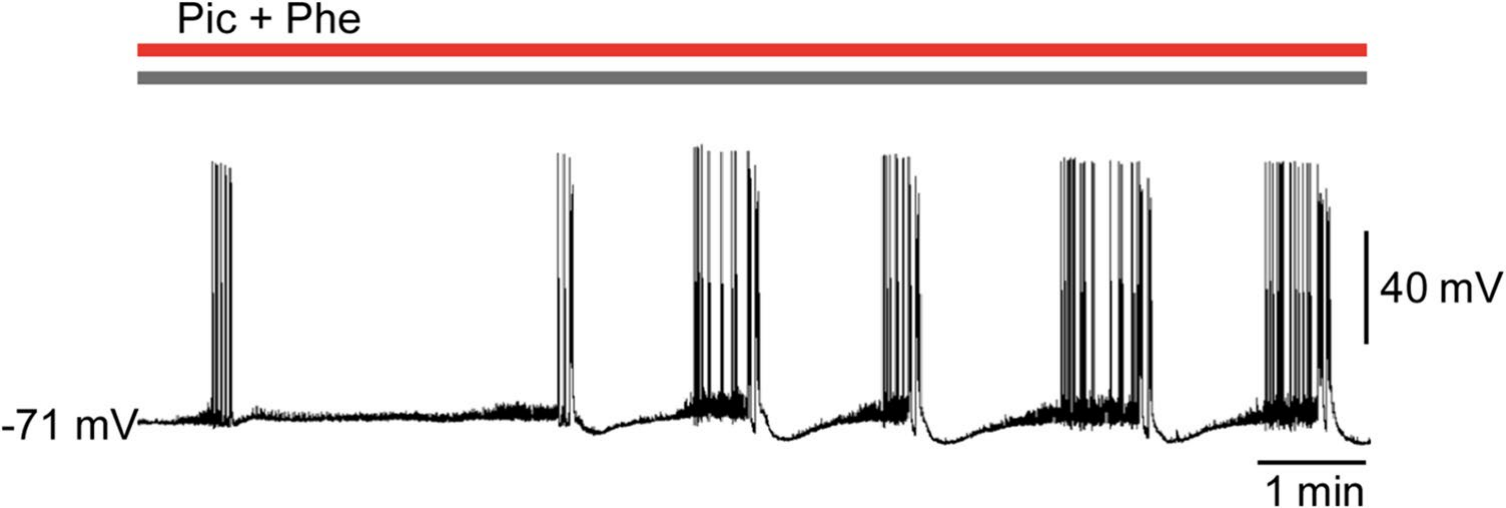
Stimulation of α_1_ adrenoceptors promotes the induction of epileptiform activities in layer 5 pyramidal cells of the medial prefrontal cortex under the suppression of inhibitory synaptic transmission. Representative trace of membrane potentials after the application of picrotoxin (Pic) + phenylephrine (Phe).

We next investigated the adrenoceptor subtypes that are involved in the NA-induced facilitation of EAs using terazosin (5 µM), atipamezole (3 µM), and timolol (10 µM), which are α_1_, α_2_, and β adrenoceptor antagonists, respectively. Addition of terazosin, but not atipamezole or timolol, to picrotoxin + NA significantly reduced the frequency of EAs (terazosin: picrotoxin + NA, 2.0 ± 0.5 events/min vs. picrotoxin + NA + terazosin, 0.4 ± 0.2 events/min, *n* = 7, *t*_6_ = 3.767, *P* = 0.0093, paired *t*-test, Fig. 4a,b; atipamezole: picrotoxin + NA, 1.6 ± 0.3 events/min vs. picrotoxin + NA + atipamezole, 1.6 ± 0.3 events/min, *n* = 7, *t*_6_ = 0.3536, *P* = 0.7358, paired *t*-test, Fig. 4c,d; and timolol: picrotoxin + NA, 2.2 ± 0.3 events/min vs. picrotoxin + NA + timolol, 1.9 ± 0.4 events/min, *n* = 6, *t*_5_ = 1.348, *P* = 0.2354, paired *t*-test, Fig. 4e,f). Moreover, we found that the addition of phenylephrine (100 µM), an α_1_ adrenoceptor agonist, to picrotoxin markedly shortened EA latency and increased the number of EAs (latency: picrotoxin, 18.3 ± 1.7 min, *n* = 6, vs. picrotoxin + phenylephrine, 1.4 ± 0.3 min, *n* = 5, *t*_9_ = 9.047, *P* < 0.0001, Student’s *t*-test; number of EAs: picrotoxin, 0.2 ± 0.2/15 min, vs. picrotoxin + phenylephrine, 17.2 ± 1.6/15 min, *t*_9_ = 11.52, *P* < 0.0001, Student’s *t*-test, Fig. 5). These findings suggest that NA promotes the induction of EAs through the activation of α_1_ adrenoceptors in the mPFC.

### NA promotes seizure induction via α_**1**_ adrenoceptors in the mPFC in vivo

**Figure 6:**
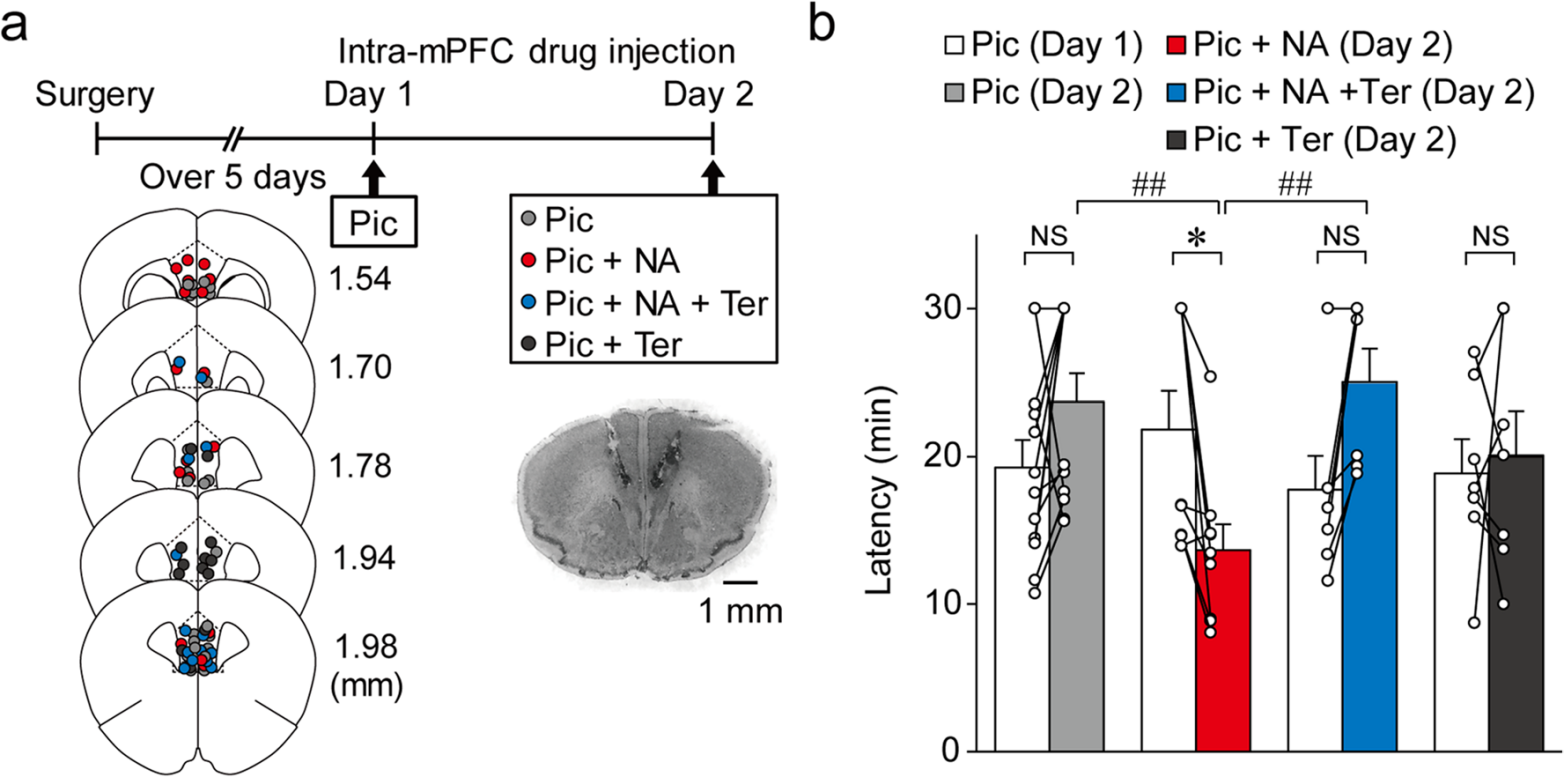
Noradrenaline (NA) promotes the induction of seizures by stimulating α_1_ adrenoceptors in vivo. (**a**) Experimental timeline, placements of infusion cannula tips, and representative micrograph: picrotoxin (Pic, light gray circles,* n* = 12), picrotoxin + noradrenaline (Pic + NA, red circles, *n* = 9), picrotoxin + noradrenaline + terazosin (Pic + NA + Ter, blue circles, *n* = 7), and picrotoxin + terazosin (Pic + Ter, dark gray circles, *n* = 8). Numbers indicate the approximate anterior–posterior distance (mm) from the bregma. (**b**) Latency of stage 3 seizures. Data are expressed as mean ± standard error of the mean. *P < 0.05, ^##^*P* < 0.01 (two-way repeated measures ANOVA with post hoc Bonferroni's test). NS, not significant.

We next investigated whether NA promotes the induction of seizures via α_1_ adrenoceptors in the mPFC. Bilateral intra-mPFC infusion of picrotoxin (0.1 nmol /side) on day 1 induced stage 3 seizures in 28 out of 35 mice within 30 min (Fig. 6a), indicating that the disinhibition of the mPFC is sufficient to induce focal mPFC seizures. On day 2, the mice were randomly divided into four groups and each group received an injection of the following drugs into the mPFC: picrotoxin only, picrotoxin + NA, picrotoxin + NA + terazosin, and picrotoxin + terazosin (Fig. 6a,b). Two-way repeated measures ANOVA revealed a significant interaction (F_3, 31_ = 5.719, *P* = 0.0031, Fig. 6b). The latency of stage 3 seizures did not show any significant change between day 1 and 2 in the picrotoxin only-injected group (day 1, 19.25 ± 1.88 min vs. day 2, 23.70 ± 1.92 min, *P* = 0.290, *n* = 12; post hoc Bonferroni's test, Fig. 6b). In contrast, intra-mPFC infusion of picrotoxin + NA on day 2 significantly shortened the latency of seizure induction when compared to picrotoxin only infusion on day 1 (picrotoxin only, day 1, 21.84 ± 2.60 min vs. picrotoxin + NA, day 2, 13.65 ± 1.76 min, *P* = 0.0230, *n* = 9, post hoc Bonferroni's test, Fig. 6b). Additionally, the latency of seizure induction in the picrotoxin + NA + terazosin-injected group on day 2 was not significantly different from that in the picrotoxin only group on day 1 (picrotoxin only, day 1, 17.76 ± 2.30 min vs. picrotoxin + NA + terazosin, day 2, 25.02 ± 2.27 min, *P* = 0.109, *n* = 7, post hoc Bonferroni's test, Fig. 6b). Similarly, the latency of seizure induction in the picrotoxin + terazosin-injected group on day 2 was not significantly different from that in the picrotoxin only-injected group on day 1 (picrotoxin only, day 1, 18.85 ± 2.33 min vs. picrotoxin + terazosin, day 2, 20.08 ± 2.98 min, *P* > 0.9999, *n* = 7, post hoc Bonferroni's test, Fig. 6b). Furthermore, on day 2, the picrotoxin + NA-injected group exhibited significantly shorter latency than the picrotoxin only-injected group (picrotoxin, 23.70 ± 1.92 min, *n* = 12 vs. picrotoxin + NA, 13.65 ± 1.76 min, *n* = 9, *P* = 0.0060, post hoc Bonferroni's test, Fig. 6b). This effect of NA was blocked by co-infusion of terazosin (picrotoxin + NA, day 2, 13.65 ± 1.76 min, *n* = 9 vs. picrotoxin + NA + terazosin, day 2, 25.02 ± 2.27 min, *n* = 7, *P* = 0.0067; post hoc Bonferroni's test, Fig. 6b). These results show that NA promotes the induction of intra-mPFC picrotoxin-induced seizures via α_1_ adrenoceptors in the mPFC.

### Restraint stress promotes the induction of seizures via α_***1***_ adrenoceptors in the mPFC

**Figure 7:**
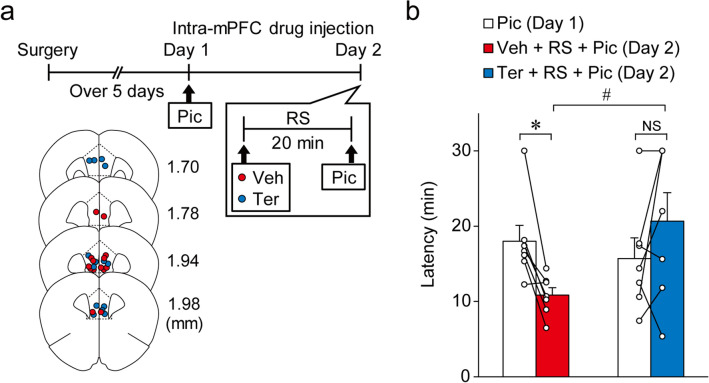
Restraint stress (RS) promotes the induction of seizures by stimulating α_1_ adrenoceptors in vivo. (**a**) Experimental timeline and placements of the infusion cannula tips: picrotoxin + restraint stress + vehicle (Pic + RS + Veh, red circles, *n* = 9) and picrotoxin + restraint stress + terazosin (Pic + RS + Ter, blue circles, *n* = 7). Numbers indicate the approximate anterior–posterior distance (mm) from the bregma. (**b**) Latency of stage 3 seizures. Data are expressed as mean ± standard error of the mean. *P < 0.05, ^#^*P* < 0.05 (two-way repeated measures ANOVA with post hoc Bonferroni's test). NS, not significant.

Because previous studies demonstrated that NA levels in the mPFC increased during restraint stress exposure^14–17^, we finally examined whether acute restraint stress promotes the induction of seizures via α_1_ adrenoceptor stimulation in the mPFC. Mice received bilateral intra-mPFC infusion of picrotoxin on day 1. On day 2, the mice were randomly divided into two groups. One group received intra-mPFC infusion of vehicle, followed by a 20-min restraint stress and then intra-mPFC picrotoxin infusion (Fig. 7a). The second group received similar treatment, with the exception of intra-mPFC infusion of terazosin instead of vehicle (Fig. 7a). Two-way repeated measures ANOVA revealed a significant interaction (F_1, 12_ = 9.851, *P* = 0.0086, Fig. 7b). The latency of seizure induction in the picrotoxin + restraint stress + vehicle group on day 2 was significantly shorter than that on day 1 (day 1, 17.99 ± 2.13 min vs. day 2, 10.84 ± 1.00 min, *P* = 0.0450, *n* = 7, post hoc Bonferroni’s test, Fig. 7b). The latency of seizures in the picrotoxin + restraint stress + terazosin group was not significantly different between day 1 and 2 (day 1, 15.70 ± 2.75 min vs. day 2, 20.67 ± 3.79 min, *P* = 0.187, *n* = 7, post hoc Bonferroni's test, Fig. 7b). However, it should be noted that the shortened latency induced by restraint stress was reversed by intra-mPFC infusion of terazosin on day 2 (picrotoxin + restraint stress + vehicle, 10.84 ± 1.00 min, *n* = 7 vs. picrotoxin + restraint stress + terazosin, 20.67 ± 3.79 min, *n* = 7, *P* = 0.0276, post hoc Bonferroni’s test, Fig. 7b). These results indicate that restraint stress promotes the induction of mPFC-originated seizures through the activation of α_1_ adrenoceptors in the mPFC.

## Discussion

The main findings of the present study were as follows: (1) bath application of NA promotes EA induction in mPFC L5 pyramidal cells via α_1_ but not α_2_ or β adrenoceptors; (2) NA-facilitated EAs are synchronously induced in the mPFC local circuit; (3) latency of intra-mPFC picrotoxin-induced seizures are shortened by co-infusion of NA, which was recovered by α_1_ adrenoceptor antagonism; and (4) acute restraint stress exposure shortens the latency of intra-mPFC picrotoxin-induced seizures via α_1_ adrenoceptors. All these findings suggest that the activation of α_1_ adrenoceptors in the mPFC by NA may contribute to the generation of stress-induced seizures in patients with epilepsy.

Electrophysiological recordings revealed that NA promotes EA induction in mPFC L5 pyramidal cells via α_1_ adrenoceptor stimulation. This result is in contrast to the finding that α_1_ adrenoceptor stimulation suppressed EA in hippocampal slices^45^. Alpha_1_ adrenoceptors are expressed in not only glutamatergic pyramidal cells but also GABAergic interneurons of the mPFC^46^. The stimulation of α_1_ adrenoceptors depolarizes GABAergic interneurons, leading to an enhanced GABAergic transmission onto mPFC pyramidal cells^47^. Considering that GABA_A_ receptors were inhibited by picrotoxin in our experimental condition, we did not observe the effect of augmented GABAergic transmission caused by α_1_ adrenoceptor stimulation. However, we have previously reported that bath application of NA induces depolarization and increases EPSCs in mPFC L5 pyramidal cells in normal ACSF^26^, indicating that α_1_ adrenoceptor stimulation in the mPFC local circuit may have an excitatory net effect even if GABAergic transmission was augmented by NA. In the hippocampal CA1, the stimulation of postsynaptic α_1A_ adrenoceptors increases firing activity of GABAergic interneurons, leading to an increased IPSC frequency in pyramidal neurons^45^. This effect of NA may suppress TLE seizures^45^. It has been reported that NA also facilitates IPSCs via stimulating α_1A_ adrenoceptors expressed at presynaptic terminals of GABAergic neurons in the basolateral amygdala^48^. However, this effect of NA is impaired by chronic stress exposure, suggesting that stress may potentiate the induction of seizures originated from the basolateral amygdala^48^. Thus, these findings suggest that the effects of α_1_ adrenoceptor stimulation on local network activity depend on the brain regions and physiological/pathophysiological conditions. Nevertheless, in the absence of GABAergic function in FLE patients, the stimulation of α_1_ adrenoceptors may facilitate hyperexcitability in the mPFC local circuit.

Glutamatergic transmission has been reported to be crucial for inducing EAs^49–51^. Consistent with this, our pharmacological examinations revealed that CNQX as well as AP-5 suppressed EAs in the mPFC slices, indicating the critical role of both AMPA and NMDA receptors in EA induction. Although NMDA receptors are blocked by Mg^2+^ at resting membrane potential, this block is removed when the membrane is depolarized. Because NA induces depolarization in mPFC L5 pyramidal cells^26^, NMDA receptor-mediated excitation accompanied with AMPA receptor-mediated transmission may contribute to the facilitation of EA induction. This is supported by a previous finding showing that the hyperactivation of NMDA receptors with free extracellular Mg^2+^ induces EA in rat hippocampal slices^52^.

Simultaneous whole-cell and field potential recordings revealed that individual EAs caused by bath application of picrotoxin + NA were time-locked to individual large field potential changes, which corresponded to population spikes, indicating the synchronism of the EAs. Additionally, EAs were observed in the mPFC-isolated slices that did not include other brain regions, suggesting that mPFC local circuitry is sufficient to induce EA. Because the mPFC contains recurrent excitatory connections^53^, activation of pyramidal cells by NA could trigger synchronous neuronal activities under the suppression of GABA_A_ receptor-mediated inhibition.

Systemic administration of convulsants, such as picrotoxin, pentylenetetrazol, pilocarpine, and kainic acid (KA), has frequently been used to generate animal models of seizures^54–57^. Additionally, the local infusion of KA into the hippocampus and amygdala, which are two major focal seizure sites, is used to generate TLE models^58,59^. However, there are few studies involving FLE models. A previous report showed that repeated intra-PFC infusion of bicuculline, a competitive GABA_A_ receptor antagonist, in adolescent rats induced focal EAs^60^. In contrast, the present study demonstrated that single intra-mPFC infusion of picrotoxin induced stage 3 seizures in most animals examined (39 out of 49 mice). However, it should be noted that we might have missed the occurrence of stages 1–2 seizures because of the paucity of observable behavior changes in stages 1–2 of the Racine scale^40,41^. Thus, single intra-mPFC picrotoxin infusion-induced seizures could be a simple and useful model of FLE. Although the local circuit activity is a primarily important for the mPFC-originated seizures, other brain regions might also be involved in seizure induction. The mPFC is known to project to and receive excitatory inputs from the thalamus that is one of the critical brain regions associated with epilepsy^61^. Thus, it is likely that abnormal excitability generated in the mPFC via stress exposure might be transmitted to this brain region, leading to the generation of epileptic seizures.

We found that the latency of stage 3 seizures induced by intra-mPFC picrotoxin + NA infusion was considerably shorter than that induced by intra-mPFC picrotoxin infusion alone. Furthermore, this shortened latency was reversed by intra-mPFC co-infusion of terazosin, indicating the involvement of α_1_ adrenoceptor stimulation in the shortening of the latency. Considering the in vitro electrophysiological data, these effects of NA and terazosin infusion on seizure latency may be attributed to the facilitation of EA generation and EA suppression by NA and terazosin, respectively, in the mPFC.

Previous studies have demonstrated the involvement of α_1_ adrenoceptors in pro- and anti-convulsant effects of NA. For example, transgenic mice with overexpression of α_1B_ adrenoceptors exhibited increased seizures^62^, whereas α_1A_ adrenoceptor agonist inhibited seizures in rat models^63^. Accordingly, it is predicted that α_1B_ adrenoceptors might have played a critical role for facilitating the seizure induction observed in the present study. However, since the function and expression of each α_1_ adrenoceptor subtype may be different in distinct brain regions^45,48^, further studies are required to elucidate the α_1_ adrenoceptor subtype(s) that are critical for facilitating the induction of mPFC-originated seizures.

Previous studies have reported that stress induces seizures^6–9^. Consistent with these findings, we demonstrated that restraint stress shortened the latency of seizure induction caused by intra-mPFC picrotoxin infusion. To our knowledge, this is the first study demonstrating that single acute restraint stress exposure affects the induction of mPFC-originated seizures. However, one study has reported that repeated restraint stress increased seizure susceptibility via the hippocampus^64^. Stress activates LC neurons, leading to increased NA release in seizure foci, such as the mPFC, hippocampus, and amygdala^14–19^. Although we did not measure the level of NA in the mPFC during the restraint stress exposure in the present study, previous studies in rats demonstrated that NA levels in the mPFC increased to approximately 150% from baseline levels during restraint stress and this increase persisted for about 30 min^14–17^, suggesting the critical role of NA for seizure facilitation. Consistent with this, we found that intra-mPFC terazosin infusion before stress exposure suppressed the latency shortening effect of stress. All these in vivo behavioral data combined with the in vitro electrophysiological results strongly suggest that stress facilitates seizure induction via elevated NA, which stimulates α_1_ adrenoceptors in the mPFC. It should be noted that restraint stress has been reported to increase the levels of not only NA but also dopamine and serotonin^17,65^. Thus, the possible involvement of these neurotransmitters in stress-induced seizure facilitation should be examined in future studies.

The mPFC can be divided into the prelimbic and infralimbic subregions. Because of a technical limitation, we could not selectively infuse drugs into each of the mPFC subregions. Further studies may be necessary to determine the role of each subregion on the stress-facilitated seizure induction.

In conclusion, our study demonstrates that NA released in the mPFC may contribute to stress-induced seizures via α_1_ adrenoceptor stimulation. Thus, α_1_ adrenoceptor blockers could be used as prophylactic and therapeutic drugs for stress-induced seizures in patients with focal mPFC epilepsy.

## Supplementary Information

Supplementary Information.

## Data Availability

The datasets generated and analyzed during the current study are available from the corresponding author on reasonable request.
