# Supplementary material for: Noradrenergic stimulation of α1 adrenoceptors in the medial prefrontal cortex mediates acute stress-induced facilitation of seizures in mice
Source: Sci Rep. 2023 May 19;13:8089. doi: 10.1038/s41598-023-35242-0 (PMC10199052; doi:10.1038/s41598-023-35242-0)
Supplement: Supplementary file 1 — Supplementary Information. [file 41598_2023_35242_MOESM1_ESM.pdf]

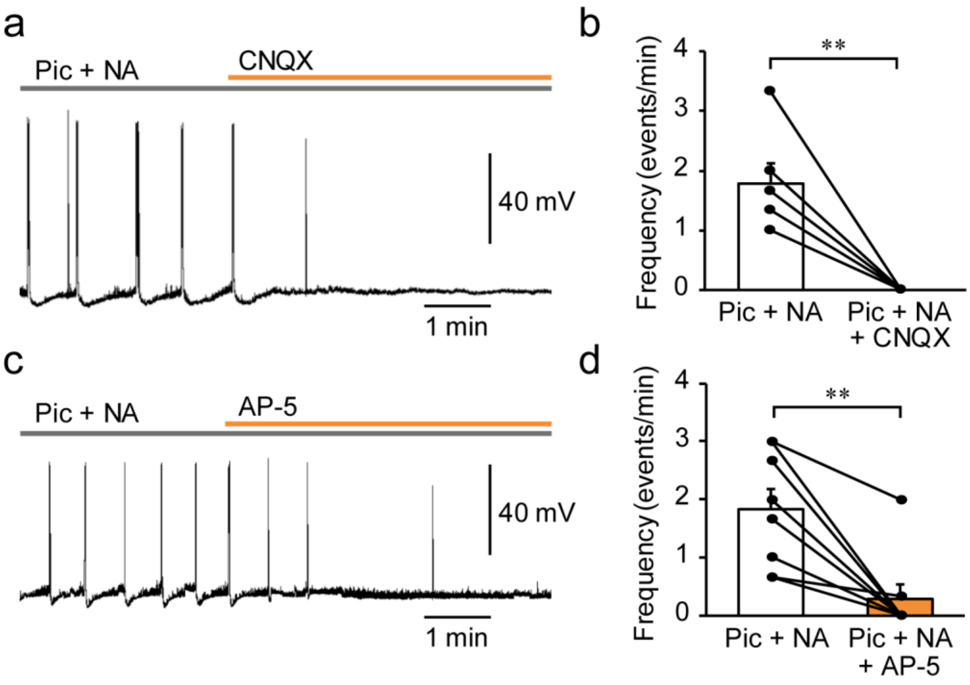

Supplementary Fig. S1. Blockade of glutamate receptors inhibits picrotoxin (Pic) + noradrenaline (NA)-induced epileptiform activities (EAs). (**a**, **c**) Representative traces of membrane potentials before and after the addition of CNQX (**a**) and AP-5 (**c**) to Pic + NA. (**b**, **d**) Summary graphs showing the effects of CNQX (**b**, $n = 6$ from 5 mice) and DL-AP-5 (AP-5; **d**, $n = 8$ from 6 mice) on EA frequency. $^{**}P < 0.01$ (paired *t*-test). Data are expressed as mean ± standard error of the mean.
